# 502 The Most Common Pathogens Isolated From Mechanically Ventilated Burn Patients With and Without Inhalation Injury

**DOI:** 10.1093/jbcr/irad045.099

**Published:** 2023-05-15

**Authors:** Kaitlin Hori, Roxana Shafiee, Haig Yenikomshian, Dianne Newman, Justin Gillenwater

**Affiliations:** University of Southern California, Los Angeles, California; California Institute of Technology, Pasadena, California; University of Southern California, Los Angeles, California; California Institute of Technology, Pasadena, California; Keck Medicine of USC, Los Angeles, California; University of Southern California, Los Angeles, California; California Institute of Technology, Pasadena, California; University of Southern California, Los Angeles, California; California Institute of Technology, Pasadena, California; Keck Medicine of USC, Los Angeles, California; University of Southern California, Los Angeles, California; California Institute of Technology, Pasadena, California; University of Southern California, Los Angeles, California; California Institute of Technology, Pasadena, California; Keck Medicine of USC, Los Angeles, California; University of Southern California, Los Angeles, California; California Institute of Technology, Pasadena, California; University of Southern California, Los Angeles, California; California Institute of Technology, Pasadena, California; Keck Medicine of USC, Los Angeles, California; University of Southern California, Los Angeles, California; California Institute of Technology, Pasadena, California; University of Southern California, Los Angeles, California; California Institute of Technology, Pasadena, California; Keck Medicine of USC, Los Angeles, California

## Abstract

**Introduction:**

The lung microbiota of patients with inhalation injury (INHI) may be distinct from those who do not have INHI. Furthermore, the lung trauma from mechanical ventilation or INHI can increase the risk of ventilator-associated pneumonia (VAP), a leading cause of death in hospital acquired infections. The purpose of this study was to identify the main pathogens isolated from mechanically ventilated patients, with and without INHI, including those that develop VAP to test the hypothesis that INHI itself influences the bacterial present in airways.

**Methods:**

Data was collected via a single institution retrospective review using the Burn Registry and adjacent health records from 1/1/2019 to 12/31/2021. VAP was defined as a hospital-acquired pneumonia that developed ≥ 48 hours of mechanical ventilation and positive culture was growth of any microorganism. Data were examined for the 3 most common pathogens cultured from respiratory samples obtained via bronchoalveolar lavage (BAL), mini-BAL, or sputum induction.

**Results:**

The 3 most common pathogens cultured from respiratory samples are presented as the percentage of the cohort in which each pathogen was identified.

In all mechanically ventilated patients (n=213), *Staphylococcus aureus* (15.9%), *Pseudomonas aeruginosa* (9.9%), and *Diphtheroid* (9.9%) were most commonly isolated. In all patients with INHI (n=78), *S. aureus* (21.3%), *Diphtheroid* (13.3%), and *gamma hemolytic Streptococcus* (10.7%) were the most common. In all patients with no INHI (n=135), *S. aureus* (14.1%), *P. aeruginosa* (10.9%) and *Diphtheroid* (7.0%) were the most common.

In those with INHI and with VAP (n=3), *S. aureus* (100%) was present in addition to *P. aeruginosa*, *Klebsiella pneumoniae*, *Acinetobacter baumannii*, and *Klebsiella aerogenes* which were each in 33% of patients.

In those with INHI and without VAP (n=75), *S. aureus* (17.3%), *Diphtheroid* (13.3%), and *gamma hemolytic Strep.* (10.7%) were the most cultured. *P. aeruginosa* was in 8% of patients.

In those with no INHI and with VAP (n=7), the most common bacteria were *S. aureus* (57.1%) followed by *Enterobacter cloacae*, *P. aeruginosa*, *gamma hemolytic Strep.*, *Diphtheroid*, *E. coli*, *A. baumanii,* and *S. pneumoniae* which were each in 28.6% of patients.

In those with no INHI and no VAP (n=128), *S. aureus* (10.9%), *P. aeruginosa* (9.4%) were found and *alpha hemolytic Strep.*, *Diphtheroid*, *K. pneumoniae* and *coagulase negative Staph.* were each in 7% of patients.

**Conclusions:**

All VAP patients had polymicrobial infections. Differences in the most common pathogens of ventilated patients with INHI versus those without INHI were seen. *P. aeruginosa* was not one of the top 3 pathogens in patients with INHI and *MRSA* was not one of the top 3 pathogens for either group. Follow up studies with a larger VAP sample size are needed to evaluate whether these trends are significant and generalizable.

**Applicability of Research to Practice:**

Understanding the microbiology of patients with and without INHI can guide targeted antibiotic treatment.

